# Like Brothers in Arms: How Hormonal Stimuli and Changes in the Metabolism Signaling Cooperate, Leading HPV Infection to Drive the Onset of Cervical Cancer

**DOI:** 10.3390/ijms23095050

**Published:** 2022-05-02

**Authors:** Matthias Läsche, Julia Gallwas, Carsten Gründker

**Affiliations:** Department of Gynecology and Obstetrics, University Medicine Göttingen, 37075 Göttingen, Germany; mlaesch@gwdg.de (M.L.); julia.gallwas@med.uni-goettingen.de (J.G.)

**Keywords:** cervical cancer, human papillomavirus (HPV), metabolism, estrogen, cytokines, microenvironment

## Abstract

Despite all precautionary actions and the possibility of using vaccinations to counteract infections caused by human papillomaviruses (HPVs), HPV-related cancers still account for approximately 5% of all carcinomas. Worldwide, many women are still excluded from adequate health care due to their social position and origin. Therefore, immense efforts in research and therapy are still required to counteract the challenges that this disease entails. The special thing about an HPV infection is that it is not only able to trick the immune system in a sophisticated way, but also, through genetic integration into the host genome, to use all the resources available to the host cells to complete the replication cycle of the virus without activating the alarm mechanisms of immune recognition and elimination. The mechanisms utilized by the virus are the metabolic, immune, and hormonal signaling pathways that it manipulates. Since the virus is dependent on replication enzymes of the host cells, it also intervenes in the cell cycle of the differentiating keratinocytes and shifts their terminal differentiation to the uppermost layers of the squamocolumnar transformation zone (TZ) of the cervix. The individual signaling pathways are closely related and equally important not only for the successful replication of the virus but also for the onset of cervical cancer. We will therefore analyze the effects of HPV infection on metabolic signaling, as well as changes in hormonal and immune signaling in the tumor and its microenvironment to understand how each level of signaling interacts to promote tumorigenesis of cervical cancer.

## 1. Introduction

The female reproductive tract is divided into an upper and a lower part. The upper part forms the uterus, the fallopian tubes and the ovaries. The lower part forms the vagina and the cervix. The cervix is divided into the endocervix (cervical canal) and ectocervix, which both can be examined by colposcopy. While the ectocervix is a stratified squamous epithelium, the endocervix consists of columnar epithelium. The squamocolumnar transformation zone (TZ) lies between the endocervix and the ectocervix. The cervix, as a mucosal epithelium, is susceptible to injury from sexual activity, which makes it sensitive to sexually transmitted infections (STIs).

**Figure 1:**
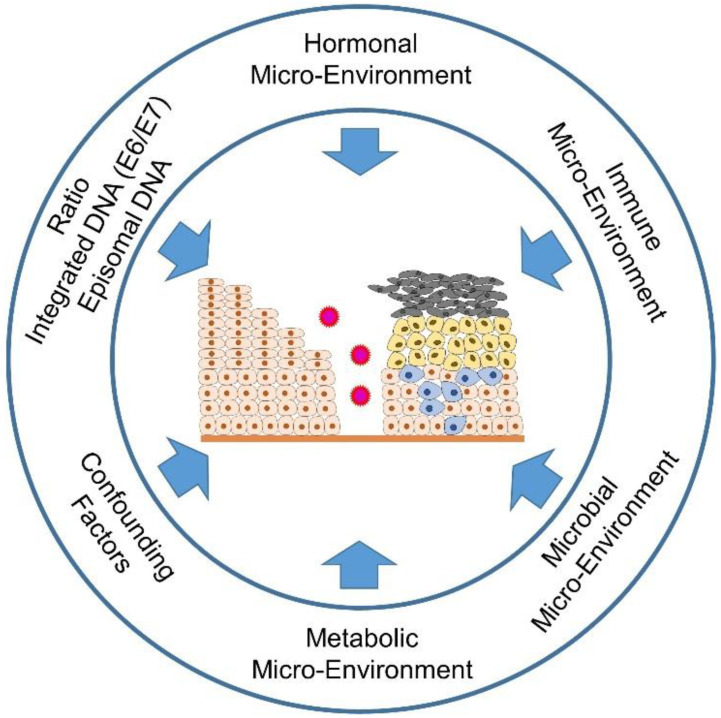
Important influences on cervical carcinogenesis.

A disease relevant to the cervix is infection with the human papillomavirus (HPV). Due to its reproductive cycle, the virus is dependent on the stratified epithelium of the cervix (Figure 1). High-risk HPV infections are responsible for 99.7% of cervical cancers, more than 90% of anal cancers, 50% of head and neck cancers, 40% of vulva cancers, as well as a minority of cancers of the vagina and penis [1,2,3,4]. HPV represents the most common sexually transmitted infection. The virus is responsible for approximately 5% of all cancers worldwide [5,6,7,8,9,10,11,12].

It is not only viruses that determine the fate of the affected cells through their development cycle, but also the microenvironment of the affected cells, i.e., the surrounding tissue, the immune cells infiltrating the tissue, but also sex steroid hormones such as estrogen and progesterone support the metamorphosis of the developing malignant cells [13,14,15]. Further influences on the fate of the developing malignancy also depend on the epidemiological and socioeconomic background of the patients since not all women have equal access to vaccination and screening programs [16,17]. Other confounding factors include age differences (pre- and postmenopausal), the time of sampling, depending on the menstrual cycle, tobacco smoking and the use of various contraceptives or other concomitant infections, such as *Chlamydia trachomatis* or human immunodeficiency virus (HIV) infections [18,19,20,21,22]. We will also discuss in this review the crosstalk between the vaginal microbiome, including HPV and other STIs, the estrobolome (capable of metabolizing estrogens via β-glucuronidases and sulfatases) and the non-estrogen-metabolizing tryptophan gut microbiota (melatonin-producing) [23,24,25]. Further, we will examine the influence of selective estrogen receptor modulators (SERMs) and selective estrogen enzyme modulators (SEEMs) or the effect of aromatase inhibitors (AIs) on estrogen signaling [15,26]. Moreover, we will take a look not only at the supportive component of sex steroid hormones in cervical carcinogenesis, but also at their (perhaps unexpected) option as a therapeutic component in the treatment of cervical lesions [13].

**Figure 2:**
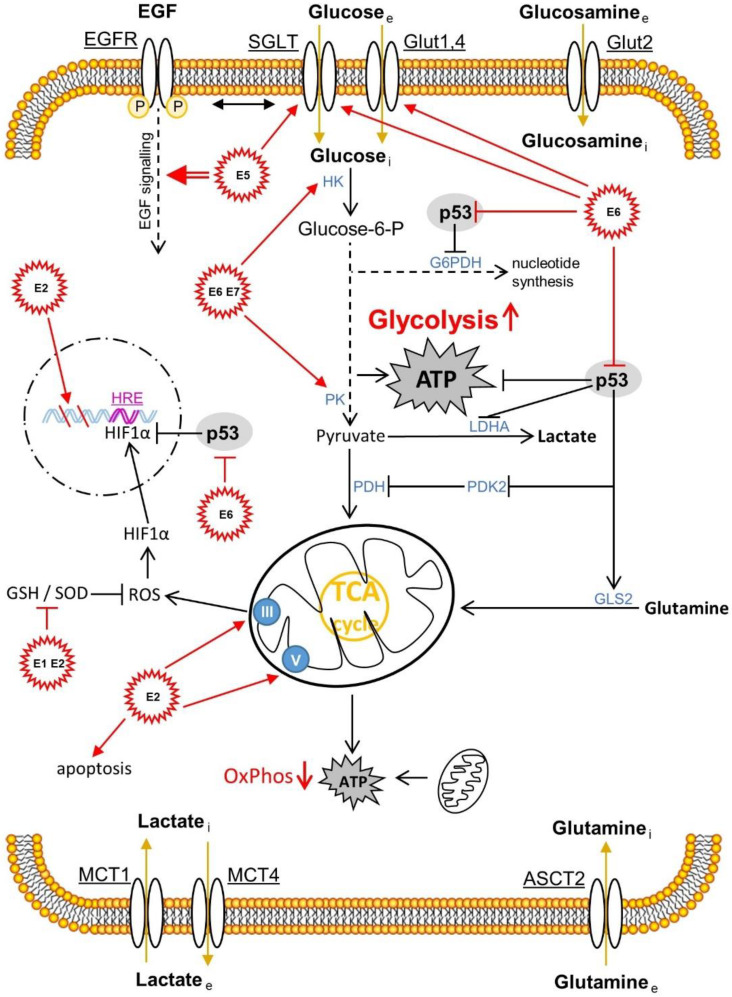
The HPV proteins E1, E2, E5, E6 and E7 and their effects on the metabolism of the transformed cervical tissue. The correlations shown are mainly presented in Section 3.1. ASCT2, amino acid transporter 2; EGF, epidermal growth factor; EGFR, epidermal growth factor receptor; G6PDH, glucose-6-phosphat-dehydrogenase; GLS2, glutaminase 2; Glut, glucose transporter; GSH, glutathione; HRE, hypoxia response element; HIF1α, hypoxia-inducible factor 1α; HK, hexokinase; LDHA, lactate dehydrogenase A; MCT, monocarboxylate transporter; OxPhos, oxidative phosphorylation; PDH, pyruvate dehydrogenase; PK, pyruvate kinase; PDK2, pyruvate dehydrogenase kinase isoform 2; ROS, reactive oxygen species; SGLT, sodium glucose transporter; SOD, superoxide dismutase. Reproduced from [30].

Besides the influences of HPV infection and the accompanying microbiota, immune [15] and metabolic [27] signaling are important determinants of patients’ health fate (pro- and anti-tumorigenic forces of the immune cells infiltrating the lesions). Higher estrogen levels generally have a positive effect on the production of antibodies and may also be associated with the differences in innate pathogen sensing and immune cell populations, thus also having a positive effect on the immune response to an infection [28,29]. On the other hand, HPV is able to suppress the arm of innate immunity via its (onco)proteins (E2, E5, E6 and E7) [30,31,32] The metabolic signaling is also influenced by the chronically altered infection and the subsequent genetic incorporation of the HPV genome coding for the oncoproteins E6 and E7 into the host DNA (Figure 2). Here, aerobic glycolysis (Warburg effect), one of the hallmarks of cancer, is increased in order to create the metabolic basis for increased proliferation, migration and metastasis. The two HPV oncoproteins E6 and E7 work cooperatively in order not only to suppress apoptosis by repressing the tumor-suppressor proteins pRb and p53, but also to increase glycolysis and inhibit oxidative phosphorylation (Figure 2) [27]. We will address the mechanisms required for this in detail later in the review.

## 2. Molecular Biological Background of HPV Infection, Structure of Viral DNA and HPV Replication Cycle in the Stratified Epithelium of the Cervix

The oncogenic process of cervix carcinogenesis requires a special structure of the mucous membranes. The border region of the squamocolumnar transition zone (TZ) between the endocervical columnar epithelium and the ectocervical squamous epithelium, with its ability to differentiate, represents an ideal location that is extremely important for carcinogenesis. In addition to the tissue’s ability to differentiate, it is the thinner layer thickness of this transition zone that mitigates the virus’ ability to penetrate the outer layers of the TZ and move forward into the basal cell layer to infect these basal cells. Small interventions, e.g., through sexual activities, enable the viruses as “stowaways” to occupy the basal cells—putative stem cells—by binding the viral V5 epitope of the virus capsid protein to the integrin receptor α6β4 of the keratinocyte [33,34,35]. This leads to the change in integrin receptor conformation, which eventually opens the “door” in the basal cell for the virus. In the basal cells, the viral DNA is released and the viral early E1 and E2 genes cooperate here in the non-reproductive cycle of the initiation phase by producing approximately 50–100 episomal copies, maintaining the viral genome [36,37,38,39,40,41,42,43,44,45,46,47,48] (Figure 1).

However, before we continue to describe the replication cycle, we would like to briefly present the individual gene sections of the virus genome and describe their function. The virus consists of a circular genome with approximately 8000 base pairs encoding six early (Early, E) and two late (Late, L) genes (eight open reading frames, ORFs) [49,50,51]. The early genes are E1, E2, E4, E5, E6 and E7; the late genes are L1 and L2. The early gene E1 ensures early replication in the initiation phase, E2 regulates the transcription of E1; but E2 also regulates its own transcription. However, the main regulatory task of E2 is the control of E6 and E7 expression. Together with E6 and E7, they are responsible for the continuation of replication and amplification in the differentiated cervical epithelium of the supra-basal keratinocytes [52,53,54,55]. The early gene E4 may be involved in the packaging of the viral DNA, and its activities take place mainly in the late life cycle of the virus and only in differentiated tissue [56]. The early gene E5 prevents the terminal differentiation of the infected keratinocyte (maintenance of the cell cycle by lengthening the S-phase), interacts with the components of the cytoskeleton, supports the assembly of the virus and, alongside its “big brothers” E6 and E7, represents the “little one” of the viral oncoproteins [57,58].

Another function of the high-risk HPV16 protein E5 is that of an oligomeric channel-forming oncoprotein from the viroporin family, which is formed in membranous environments [59]. This hexameric E5 channel mediates effects on cell signaling pathways such as epidermal growth factor receptor (EGFR) signaling, which it enhances, and keratinocyte growth factor (KGFR) signaling, which it, in turn, inhibits. While cell cycle progression is promoted via the EGFR pathway, the KGFR pathway impairs the differentiation of keratinocytes and thus leads to increased proliferation [60].

The HPV18 protein E6 can also accelerate the life cycle of the HPV virus in both differentiated and undifferentiated keratinocytes. Here, the signal converter and activator of transcription 3 (STAT-3) acts as an antagonist of STAT-1 signaling, which also suppresses the immune response. STAT-3 activation via dual phosphorylation via Ser727 and Tyr705 requires members of the Janus kinases and the mitogen-activated protein kinase (MAPK) family. E6 mediates the STAT-3-induced transcription of genes such as cyclin D1 or Bcl-xL, which promote cell cycle progression or prevent apoptotic cell death. Thus, STAT-3 makes a crucial contribution to cervical carcinogenesis [61]. The pro-inflammatory cytokine interleukin 6 (IL-6) mediates STAT3 phosphorylation in an autocrine and paracrine manner. The production of IL-6 itself requires the phosphorylation of the transcription factor nuclear factor ‘kappa light chain enhancer’ of activated B cells (NFκB) and the involvement of the small GTPase Rac1. Active STAT-3 thus drives a gene expression program important for cell proliferation and the survival of cervical cancer cells [62].

E6 and E7 are the two major HPV oncoproteins; the E6 protein, by binding and deactivating the tumor suppressor protein p53, and the E7 protein, by binding the retinoblastoma protein pRb, ensure their transforming capacities [63,64,65,66,67].

How HPV oncoproteins E5, E6 and E7 drive the malignant transformation of cervical keratinocytes, prolong their cell cycle progression, disrupt keratinocyte differentiation, prevent host cell apoptosis and create an environment favorable for the virus to prevent an adequate immune response, supporting HPV infection to persist, explains a recently published review article [68].

Due to the low expression level of HPV proteins in the initiation phase within the undifferentiated basal cell layer, the virus likely evades immune recognition in order to maintain the persistent infection. The highly immunogenic viral L1 and L2 capsid proteins, on the other hand, are expressed late in the reproductive cycle in the outermost layers of the squamocolumnar TZ of the cervical epithelium, in areas that are soon passively sloughed off with the uppermost layers of the cervical epithelium by mechanical abrasion, and hence cause no cell lysis or necrosis. This delay in the expression of the L proteins is achieved by controlling various levels of regulation. These include the splicing regulation of the donor and acceptor, the shift of a polyadenylation signal from an early to a late signal and the integration of rare codon sequences [69]. Thus, the virus can spread further—unnoticed by the immune system—and is supported by further sexual activities [70,71,72,73].

In order to complete the replication cycle, there is an important hurdle that needs to be cleared. Normally, when keratinocytes reach the differentiation phase, replication goes to rest. This leads to cell cycle arrest of the host cells. The virus now has to have the host cell restart replication and therewith the activity of the replication enzymes if it wants to benefit from it, since it does not code for its own replication enzymes and is dependent on those of the host cell. For this purpose, the HPV E7 oncoprotein disrupts cell cycle checkpoints by high-affinity interactions with various family members of the retinoblastoma (pRb) family and displacement of the E2F transcription factor from the enzyme complex. This activates the S-phase genes and returns the infected keratinocytes to the cell cycle [45,74].

The resumption of cell cycle progression thus also leads to increased transcription of the genes E1, E2 and E1^E4 (E1 amino acids connected to the E4 ORF) and the E5 oncogene [75,76,77]. This results in a strong increase in replication, which increases the number of episomal virus DNA copies to several thousand per host cell [78]. The genes E1^E4 and E5 are only transcribed in the later keratinocyte differentiation phase with the beginning of the later functions of the virus [79,80,81]. The E7 oncogene, on the other hand, activates the Ataxia telangiectasia mutated (ATM) damage pathway, which is important for replication at the appropriate foci and for amplification [82].

### 2.1. Viral Proteins Determining Host Cell Fate in Terms of Immune Evasion and Duration of HPV Infection and Transformation

The early high-risk (HR) viral oncoproteins E5, E6, and E7 cooperatively suppress both innate (nonspecific) and adaptive (specific) immune responses. However, the late minority L2 protein also appears to affect the arm of innate immunity negatively via blocking the maturation of Langerhans cells (LCs). Indeed, in the vast majority of cases, infection with HPV leads to the elimination of the infection after the appearance of lesions caused by the infection, since there is an adequate immune response in most cases [83]. Only in the case of a continued infection, which may last for several years, and which is perhaps also supported by an accompanying “pathological” microbiota [84], the immune system no longer manages to completely eliminate the infection, which increases the risk of transformation of the cervical epithelium [85,86].

The interferon response pathway also belongs to the innate immunity arm [73,87]. Many of its targets are suppressed via interaction with the E6 and E7 oncoproteins. For example, E6 binds to interferon regulatory factor 3 (IRF-3) and inhibits its function. The same happens with the interferon regulatory factor 1 (IRF-1) through the E7 oncoprotein [83]. However, the HR E6 and E7 oncoproteins also suppress target genes of the interferon signaling pathway such as the signal converter and activator of transcription 1 (STAT-1) [88,89]. On the one hand, RNA protein kinase R (PKR) is suppressed via the interaction with p-bodies [90], while on the other hand, tyrosine kinase 2 (TYK-2), a member of the JAK family, which contributes to STAT-1 activation, is blocked [83,91]. Toll-like receptor 9 (TLR-9), also named the CD289 differentiation cluster, recognizes pathogen-associated molecular patterns (PAMPs). PAMPs, in this case, unmethylated CpG oligonucleotides, i.e., DNA, characterize a large number of microorganisms. TLR-9 expression levels are now suppressed through the interaction with the HR E6 and E7 oncoproteins [92]. Thus, this interaction through this pathway suppresses immune recognition within the keratinocytes of the cervical epithelium, thereby causing immune evasion. The targets of the host’s interferon response are not the only ones being blocked. The constitutive interferon κ expression itself, and thus the subsequent gene induction, are also suppressed [93].

**Figure 3:**
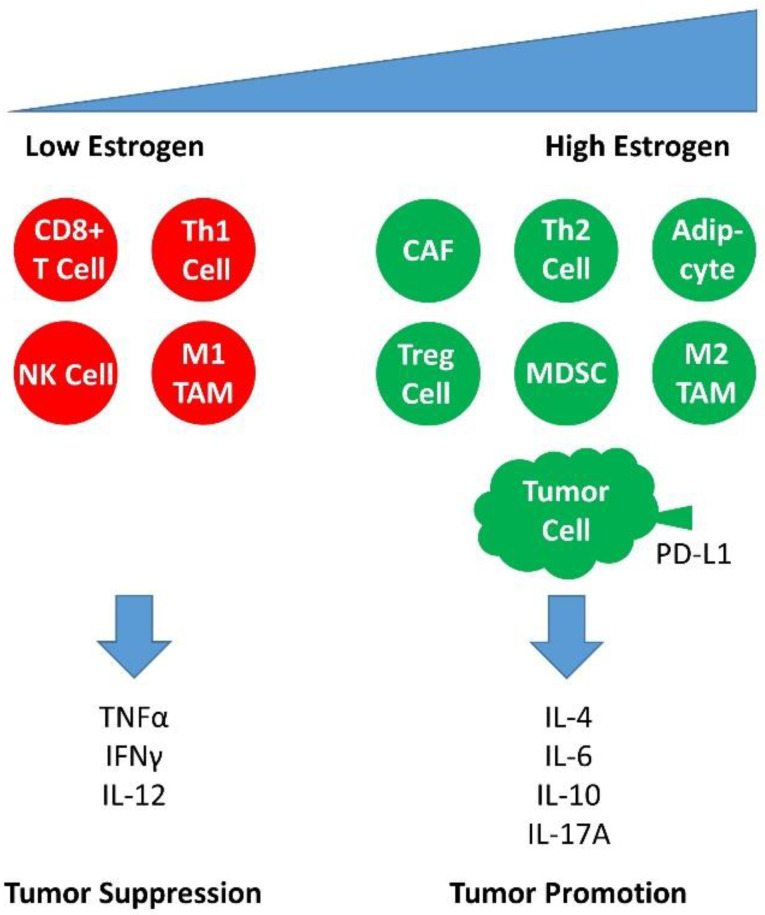
Pro- and anti-tumoral effects of members of the tumor microenvironment on the immune microenvironment of cervical intraepithelial neoplasia and malignancy, respectively, as a function of estrogen concentration. Depending on the strength of estrogen signaling, cells of the innate and adaptive immune response are modulated according to the communication between them and depending on their cytokine levels in such a way that they support either tumor suppression or progression. This battle between the subtypes of immune cells determines whether the tumor progresses or is eliminated by the immune system. The tumor-suppressive arm consists of CD8+ (cytotoxic) T cells, Type 1 T helper cells (Th1 lymphocytes), natural killer (NK) cells, type 1 tumor-associated macrophages (M1 TAMs). The tumor-promoting arm consists of cancer-associated fibroblasts (CAFs), Type 2 T helper cells (Th2 lymphocytes), adipocytes, regulatory (suppressive) T cells (Tregs), MDSCs, type 2 tumor-associated macrophages (M2 TAMs), Tumor cells. IFNγ, Interferon gamma; TNFα, tumor necrosis factor alpha; IL, Interleukin; PD-L1, Programmed cell death 1 ligand 1.

As a rule, infection is associated with an inflammatory reaction and a consequently increased production of cytokines [94]. However, when the keratinocytes of the cervical epithelium are infected by HPV, the production of inflammatory cytokines such as interleukin 1 (IL-1), IL-6, IL-12, interferon γ (IFNγ) and tumor necrosis factor alpha (TNF-α) is reduced [95,96,97], and the production of anti-inflammatory cytokines such as interleukin 4 (IL-4), IL-10, IL-13 and transforming growth factor beta (TGF-β) increased [92,95] (Figure 3). This has implications for the activities of immune cells of the innate immune system, such as Langerhans cells (LCs) and dendritic cells (DCs), as well as cells of the adaptive arm of immunity, activated cytotoxic CD8+ and CD4+ T lymphocytes. As a result, fewer of these immune cells infiltrate the infected keratinocyte host tissue of the cervical epithelium, leading to immune evasion and the persistence of HPV infection [73,98,99]. In addition to the HR HPV E6 and E7 oncoproteins, the HPV L2 protein may also affect LC development and mediate decreased levels of E-cadherin and the subsequent attenuation of LC maturation, migration and secretion of cytokines by LCs. This strongly inhibits the crosstalk between the infected keratinocytes of the cervical epithelium and the LCs infiltrating them [92,100].

The class 1 major histocompatibility complex (MHC I), another important building block of the adaptive immune response, is expressed on the surfaces of almost all cells. It provides the presentation of antigens to the CD4+ and CD8+ T lymphocytes that recognize them via a corresponding T cell reaction. The HR HPV proteins E5, E6 and E7 are able to suppress the expression levels of MHC I [101,102]. E7 also down-regulates the antigen peptide transporter TAP, which is associated with antigen processing [92]. The natural killer (NK) cells belong to the arm of the innate immune response. Their function is also disturbed by the HPV oncoproteins, and they can no longer detect the loss of MHC I expression on the keratinocytes [83,101]. Thus, HPV suppresses both the innate and the adaptive arm of the immunity of the host cells via the effects of their oncoproteins, and is thereby able to evade immune recognition and persist in the host tissue. At the same time, the processes described also support the malignant transformation of the cervical epithelium.

### 2.2. The Demand for Oxygen and Nutrients within HPV-Infected Lesions Promotes Angiogenesis

Persistent HPV infections that are not cleared by an inefficient immune system can, after a few years, create benign or malignant lesions called cervical intraepithelial neoplasias (CINs). This accumulation of altered cells either is cleared away by the immune system or may develop further into carcinoma. In both benign and malignant lesions, an area of lack of oxygen and nutrients develops in the center of the developing neoplasia due to the competition of cells for oxygen and nutrients. This normally leads to cell death via the initiated cell cycle arrest. A means of choice for stressed cells is angiogenesis. Sprouting and fission give rise to new blood vessels from existing vessels, which halts cell death through a better supply of nutrients and oxygen [103,104,105]. Oxygen starvation in the hypoxic core within the lesions also leads to the up-regulation of genes associated with angiogenesis, glycolysis and erythropoiesis via the transcription factor, hypoxia-inducible factor 1 (HIF-1). These genes are controlled and up-regulated via the hypoxia response element (HRE) and ensure cell survival within the hypoxic tissue [106]. Both benign and malignant lesions thus benefit from the compensatory response due to the deficiency conditions. Not only the high-risk (HR) E7 oncoproteins, but also the low-risk (LR) E7 oncoproteins, are able to increase HIF-1α and its gene target levels. In the process, histone deacetylases (HDACs) are displaced from the histone complex of HIF-1 [107,108,109,110,111,112]. Only neo-angiogenesis provides the conditions necessary for successful malignant transformation of the cervical epithelium.

### 2.3. The Effects of HPV Oncoproteins on Cellular Metabolism within the Cervical Intraepithelial Lesions

HPV infection is eliminated within 2 years in 90% of cases, even in cases of high-risk HPV, but only if the tandem genome coding for the E6 and E7 oncoproteins does not integrate into the human genome, and only if the patient’s immune system is competent [113]. In most cases, there is no malignant change in the cervical epithelium. Failure to clear the virus from HR HPV lesions of the cervix for many years, perhaps as a result of a compromised, incompetent immune system, may lead to genomic instability due to HR E6 and E7 oncoproteins interacting with their target genes sites. This can result in segments of viral DNA, namely the E6 and E7 ORFs, inserting into unstable regions of host DNA and causing integration-induced hyper-regulation of the E6 and E7 sections. As a result, mutations could arise and accumulate, which would ultimately promote malignancy [114,115]. The E1 and E2 genes, which regulate the rate of transcription of the viral genome, may be disrupted upon integration into host DNA. The E2 gene can be damaged through methylation of locus control region (LCR) binding sites. The loss of these transcription control instances then leads to hyper-expression of the integrated E6/E7 tandem [114,116]. This often results in so-called super-enhancer-like elements [117]. The ratio of integrated HPV DNA to episomal DNA increases over time, which can accelerate the development of the lesion into a premalignant precursor and further into cervical carcinoma (CxCa) [118]. Ultimately, after full integration of the E6 and E7 ORFs, no viral episomes are present in the host cells, and with the initiation of malignant development, the virus’ own reproduction ends.

If the integration of the HR HPV E6 and E7 sections into the host DNA leads to the suppression of the tumor suppressor proteins pRb and p53, this not only leads to the suppression of apoptosis. The E6 oncoprotein blocks TIGAR via interaction with p53. TIGAR regulates glycolysis and apoptosis. On the one hand, glycolysis is activated, and at the same time, oxidative phosphorylation (OxPhos) is suppressed. The latter occurs via the assembly protein of cytochrome c oxidase (SCO2) [119,120]. Thus, via binding to p53, the HPV16 E6 oncoprotein promotes glycolysis, an eminently important source of fast energy and intermediate metabolites for cell formation in cancer cells [121]. Nevertheless, E6 also binds to c-Myc, one of the most important transcription factors of all, and a key component that is a hallmark of cancer development. In addition to the function of ubiquitin-dependent degradation of the tumor suppressor protein p53, E6 forms a complex with Myc that is able to bind to and transactivate the catalytic subunit of the human telomerase reverse transcriptase (hTERT) promoter. Through this process, the E6 protein is able to modulate cell proliferation and differentiation by binding to Myc [122].

Many important enzymes that determine the rate of glucose breakdown and its utilization, such as α-enolase 1 (ENO 1), hexokinase 1 and 2 (HK 1/2), lactate dehydrogenase A (LDHA), phosphofructokinase 1 and 2 (PFK 1/2) and the glucose transporter proteins GLUT1 to GLUT4 increase aerobic glycolysis due to their aberrantly increased expression levels [122,123]. This is also known as the “Warburg effect”, one of the most important cancer hallmarks, and was first published by Otto Warburg in 1924 [124,125,126]. When the virus DNA sections are integrated within the host DNA, primarily fragile sites that co-localize with the myc locus are used [114,127,128,129], which, on the other hand, is also not a universal phenomenon associated with HPV integration.

HR HPV E6 promotes enhanced activation of the phosphoinositide-dependent protein kinase 1 (PDK1)- and mammalian target rapamycin complex 2 (mTORC2)- signaling pathways via the phosphoinositide-3-kinases (PI3Ks)/protein kinases B (AKT, PKB) pathway and the activity of the mTORC1 signaling pathway [130]. mTORC1 is regulated by growth factors, and as a metabolic sensor, it also perceives the oxygen supply of the tissue in which the hypoxia-inducible factor 1 (HIF-1) transcription factor accumulates in the cell nucleus and up-regulates the expression level of genes via hypoxia-responsive elements (HREs) that boost metabolism. This includes, for example, the glucose transporter protein GLUT-1 [131]. In this context, the E6 oncoprotein of HR HPV16 also increases the expression of pyruvate kinase 2 (PKM2), an important co-activator enzyme of HIF-1 [131] (Figure 2).

In oral squamous cell carcinoma, the E7 oncoprotein of HR HPV16 also promotes HIF-1 activation [132]. Via the activity of the epidermal growth factor receptor (EGFR) signaling and the subsequent signaling via the extracellular signal-regulated kinase 1 and 2 (ERK1/2) and AKT (PKB), the viral protein E5, referred to as a “small oncoprotein”, activates the Warburg effect [133,134]. Similar mechanisms may also apply to cervical carcinoma.

GLUT transporter proteins transport glucose, various alternative monosaccharides, polyols and other small carbohydrates across the cell membrane. This process is operated by means of so-called facilitated diffusion. This passive transport process contrasts with active transport processes such as secondary active transport by sodium-glucose-linked co-transporters (SGLTs) [135,136,137,138,139,140]. Cancer cells require many nutrients for their aberrant growth compared to non-malignant cells. To avoid this bottleneck in the supply of the developing malignancy, cancer cells use these SGLT transporters when high energy (ATP) and metabolites are required but the supply of glucose is deficient. For example, the HR HPV18 oncoprotein E6 is able to up-regulate the expression levels of the SGLT1 glucose transporter [135,136,137,141,142]. The up-regulation of EGFR expression is more widespread in cancer cells. EGFR is also co-localized to the SGLT transporter. This leads to the stabilization of SGLT and increased glucose influx [135,136,137,142]. The same applies to cervical carcinoma, mediated via the HR HPV16 E6 oncoprotein. Here, E6 represses the tumor suppressor protein p53, which, under normal circumstances, suppresses the expression of the GLUT transporter, which in turn greatly increases the influx of glucose [143]. In lung cancer, the HR HPV16 E6 oncoprotein impedes the interaction between the von Hippel–Lindau (VHL) tumor suppressor protein and HIF1α and, via activation of the latter, increases the expression levels of GLUT1 and thereby aerobic glycolysis by an increased influx of glucose [144,145]. In primary oocytes of the African clawed frog *Xenopus laevis*, co-expression with the HR HPV18 E6 oncoprotein significantly increases membrane SGLT1 expression [141].

The HR HPV E6 and E7 oncoproteins act on many levels of metabolic signaling. This includes not only the glycolytic pathway but also the citrate cycle [146]. In the glycolytic pathway, inactivation of the tumor suppressor protein p53 increases the levels of nucleotides synthesized in the pentose phosphate pathway (PPP) via glucose-6-phosphate dehydrogenase (G6PD(H)). As previously described, P53, an antagonist of G6PD(H), a key enzyme in PPP, is bound by the HPV oncoprotein E6 and converted to degradation [147]. The E6 oncoprotein up-regulates pyruvate dehydrogenase kinase 2 (PDK-2) and consequently down-regulates pyruvate dehydrogenase (PDH). This suppresses acetyl-coenzyme A (acetyl-CoA) levels and causes the metabolism to drift away from oxidative phosphorylation toward increased lactate production, a hallmark of cancer and consistent with the Warburg effect. Degradation of the tumor suppressor protein p53 also decreases glutaminolysis by down-regulating levels of glutaminase 2 (GLS2). This results in reduced levels of α-ketoglutarate, an intermediate in the cellular ATP energy metabolism of the citric acid cycle. As a result, the function of the citric acid cycle is also suppressed, which in turn is characteristic of cancer.

It was previously discussed that pyruvate kinase 2 (PKM2) is a co-activator of HIF-1 [131]. PKM2 is also often referred to as tumor M2 pyruvate kinase. It represents the dimeric fetal form (M2) of pyruvate kinase. Compared to the tetrameric adult form of pyruvate kinase 1 (PKM1), it has greater enzymatic activity in the metabolism arm of glycolysis [121,148]. In mouse embryonic fibroblast cells (NIH-3T3), direct binding of the HR HPV16 E7 oncoprotein to PKM2 mediated the dimerization of the latter. The dimerized PKM2 increases the activity of glycolysis and glutaminolysis and the production of nucleotides. There is also increased consumption of glucose and serine, and increased cell proliferation of the NIH-3T3 cells. In addition, the crosstalk between glucose and glutamine metabolism and glutaminolysis itself is enhanced, which is revealed in the correlation between alanine and lactate production [149]. The HR HPV E6 and E7 oncoproteins ultimately lead to the progression of malignant transformation that is associated with the isoform change from M1 to M2 (tumor M2-PK) and the metabolic shift towards glycolytic cancer metabolism [150]. Lactate dehydrogenase A (LDHA) is another important enzyme that shifts the metabolism with the main product lactate towards aerobic glycolysis (Warburg effect). The metabolized sugars are not completely metabolized via the citric acid cycle and oxidative phosphorylation (OxPhos); instead, the pyruvate is reduced to lactate in a reversible reaction [121].

The HPV E6 oncoprotein also shows another effect, mediated by the degradation of the tumor suppressor protein p53, on short non-coding microRNAs (miRNAs) [58,151]. In a special case, the microRNA 34a (miR-34a), which is up-regulated by p53, represents a tumor suppressor that negatively regulates the Warburg effect [152]. The expression level of miR-34a is down-regulated via the degradation of p53 through interaction with the E6 protein, and the lactate level is increased via the increased activity of lactate dehydrogenase A (LDHA) and thus the Warburg effect [153].

The tumor suppressor protein p53 has diverse effects. To provide another example, it is able to regulate the expression of mitochondrial proteins such as the assembly protein of cytochrome c oxidase 2 (SCO2, cytochrome c: Oxygen oxidoreductase (COX) or complex IV) [120], apoptosis-inducing factor (AIF) [154], a positive intrinsic regulator of apoptosis and ferredoxin NADP+ reductase (FNR, NADPH: Adrenodoxine oxidoreductase, FDXR) [155]. These enzymes are extremely important for correct mitochondrial function (oxidative phosphorylation, OxPhos) and the integrity of the mitochondrial structure [156].

The HPV E2 protein, as the main regulator of viral DNA transcription, regulates, cooperatively with the E1 protein, the transcription and thus the translation of the HR HPV E6 and E7 oncoproteins. The E2 protein migrates continuously between the nucleus and the cytoplasm. The E2 protein in the cytoplasm promotes apoptosis [157]. It provokes mitotic DNA strand breaks and destabilizes the chromosomes. This is considered the cause of the integration of the HPV E6 and E7 DNA segments into the host genome [158]. However, the E2 protein also affects cancer metabolism by binding to the mitochondrial membrane, and reductive oxygen species (ROS) are released via changes in the crystal structure of the mitochondrial membrane proteins. The released ROS stabilize the nuclear HIF complex and thereby increase the rate of glycolysis [159]. While the morphology of mitochondrial protein crystals is regulated by the F_0_F_1_-ATPase (Complex V) [160], the proteins of Complex III mediate the mitochondrial release of ROS [161]. The E2 protein modulates the latter. ROS act as secondary messengers and modulate cellular homeostasis and cell proliferation and differentiation. Another important effect of ROS is its potential to alter mitochondria or damage nucleic acids, fats or proteins due to aberrant glycolysis via oxidative stress (OS) [121]. The cell is able to effectively counteract and mitigate the OS via molecules of various antioxidant systems [162]. These include, among the peroxidases (Prxs), primarily the systems of glutathione peroxidases (GSHPX), reduced glutathione (γ-L-glutamyl-L-cysteinylglycine, GSH), catalases (CAT), superoxide dismutases 1, 2 and 3 (SOD1/2/3) and the thioredoxins (TRDX). GSHPX and SOD1/2 are down-regulated, for example, by the co-expressed proteins of HR HPV 16/18 E1 and E2, which damages the DNA through the unhindered release of ROS [163].

Recently, it was found that 17β-estradiol (E2) altered the properties of mitochondria. Modulations of their permeability, aberrations in calcium signaling and reduction in levels of nitric oxide (NO) and lipid oxidation led to a reduction in oxidative phosphorylation (OxPhos) and increased glycolysis (Warburg effect). Genes of the electron transport chain (ETC) of the mitochondria, OxPhos components and the glycolytic degradation pathway, including the pentose phosphate pathway (PPP), were all differentially expressed. Therefore, metabolic signaling was deregulated by E2 in cervical carcinoma cell lines of SiHa, HeLa and C33a towards aerobic glycolysis [164].

In HeLa cervical carcinoma cells, it was found that steroid sulfatase (STS), which, in addition to estrone sulfate (E1-S), also converts other steroid sulfates such as DHEA-S to DHEA (dehydroepiandrosterone, prasterone), drives the Warburg effect. The up-regulation of the most important rate-determining enzymes in glycolysis, such as hexokinase 2 (HK2) and type M2 pyruvate kinase (PKM2, also known as tumor M2-PK), is promoted. Prasterone (DHEA), the most abundant steroid hormone in the human body, synthesized in the liver, is further metabolized to testosterone, which in turn can be converted to estradiol (E2). STS mediated its effect by increasing both the transcription and translation activity of the hypoxia-inducible factor (HIF-1α), which could also be shown in vivo in the lung tissue of transgenic mice and also increased HIF-1α-, HK2- and PKM2 levels here. This further led to increased lactate production with simultaneous repression of mitochondrial activity, which was indicated by a reduction in the oxygen consumption rate (OCR). However, DHEA also achieved the same effects as STS, but not the sulfated form, DHEA-S [165].

## 3. The Effects of the Hormone Microenvironment on Cervical Carcinogenesis

### 3.1. General Information about Sex Steroid Hormones, Their Biosynthesis and Their Localization in the Female Body

Of the estrogens circulating in the female body, 17β-estradiol (also referred to as estradiol or E2) is the predominant form of the sex steroid hormone. It plays an important role in both sexes. In addition to the discussed functions in the menstrual cycle, the predominant estrogen is present at high levels during the reproductive phase in women [166]. At the beginning of menopause, the production of estrogen drops significantly and thus leads to well-known symptoms in women in this phase of life [166]. Bone health, heart health, female fertility, various regulatory effects on glucose metabolism, the maintenance of immune function, nerve function and the role of estrogens in the treatment of many pathologies are also modulated by estrogen signaling [114,127,128,129,166,167,168,169,170,171,172,173,174,175,176,177,178,179,180,181]. The estrogen circulating in the woman’s body acts in an endocrine, paracrine or intracrine manner and binds to the three most important of its receptors, the estrogen receptor α (ERα), the estrogen receptor β (ERβ) or the G protein-coupled estrogen receptor 1 (GPER1) [182,183].

**Figure 4:**
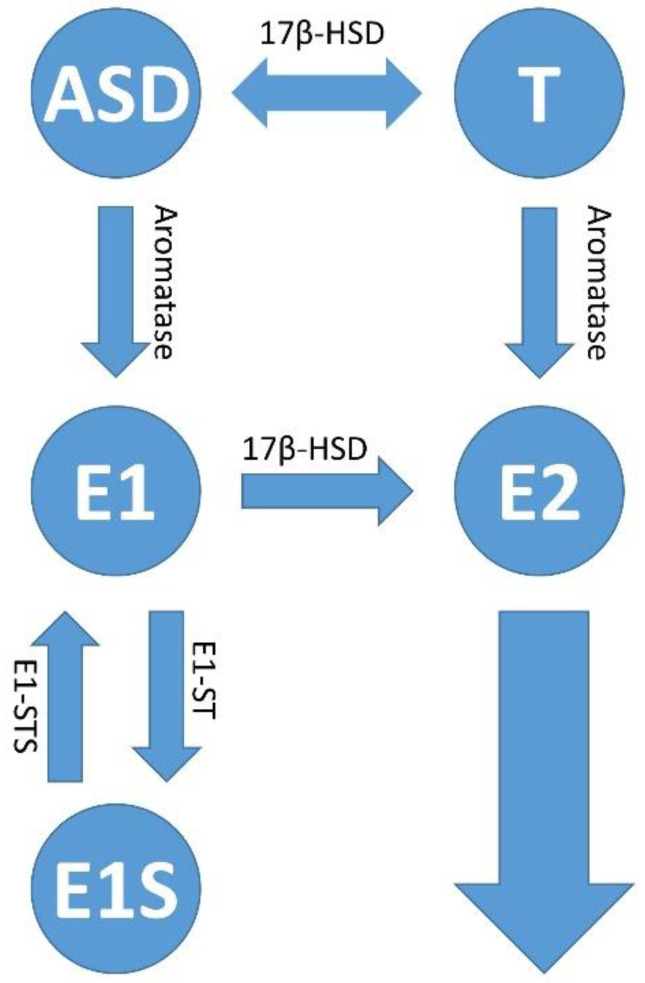
Estrogen synthesis pathways. In addition to estradiol (E2), estrone (E1) is also formed in the human body when androstenedione (ASD) is aromatized. This mainly happens in postmenopausal women. Estrone (E1) and/or estradiol (E2) are delivered to the tumor and its microenvironment via the bloodstream; or there is increased local production and retention of the hormones within the tumors. The latter occurs via increased activity of the cytochrome P450 enzyme, aromatase (CYP19A1), which converts androstenedione (ASD) to E1 and testosterone (T) to E2. The enzymatic activity of estrone (E1) sulfatase converts estrone sulfate (E1S) to estrone (E1); and 17β-hydroxysteroid dehydrogenase type 1 (17β-HSD1) activity is responsible for the conversion of E1 to E2, and androstenedione (ASD/AE) to testosterone (T), respectively. The sulfation of estradiol (E2) leads to the inactive form of estrogen, estrogen sulfate (E1S) via the activity of estrone sulfotransferase (E1-ST).

Most estrogen is formed in the ovaries of women, but also in adipose tissue and the adrenal glands in women and men [183,184,185,186]. Androstenedione (ASD), which is also well-known in the bodybuilder scene, can be formed in adipose tissue from low-density lipoprotein (LDL) cholesterol via various enzymatic steps. ASD can then be metabolized to any of the steroid hormones in addition to estrogen [183,187,188]. In addition to the gonads (ovaries), estradiol is also synthesized via Peyer’s patches in the intestine [187]. In 1645, Italian anatomist and surgeon Marco Aurelio Severino referred to these structures in the gut as “lymphoid follicles.” Just 32 years later, they were named Peyer’s patches after the Swiss pathologist Johann Conrad Peyer, who described these intestinal structures in detail. The intestines, but also all other human mucous membranes, are colonized by a large number of microorganisms. These symbiotic but also pathogenic microorganisms form, among other things, the intestinal flora (intestinal microbiota), a community of bacteria, primordial bacteria, fungi, protozoa and even viruses [189]. The habitat of the microorganisms, their genome, together with the neighboring microenvironment, are called the microbiome [190]. The number of bacteria colonizing human mucous membranes and other epithelia is estimated to be as large as there are cells in the human body [191]. However, compared to humans, the bacterial community has a metagenome that is 100 times larger [192]. What is special about the intestinal microbiome is that, as a so-called estrobolome [15,193], in contrast to the non-metabolizing tryptophan microbiome, it is able to regulate the free estradiol level in the intestine and in the mucosal epithelia outside the intestine. In addition to estradiol (E2), estrone (E1) is also formed in the human body when androstenedione (ASD) is aromatized. This mainly happens in postmenopausal women. If the woman is pregnant, her body mainly produces the third form of estrogen, estriol (E3) [194] (Figure 4).

Estrone (E1) and estradiol (E2) are the starting molecules of estrogens, which are converted into metabolites in the liver and occur as hormones with different potency [195]. Subsequently, the starting molecules and their metabolites are converted to glucuronides and sulfates by glucuronidation or sulfation in order to be excreted via the bile, the kidneys or the intestines [196]. Just as estrogens are conjugated, they can also be de-conjugated by bacteria in the stomach and intestines. This occurs via β-glucuronidases (GUSB) or β-glucosidases from the class of glycosidases [197]. The bacteria in the intestine recycle the estradiol, and this can possibly fuel neoplasia, the development of which depends on estrogen signaling. The intestinal bacteria de-conjugate the bound estrogens and release them via the secretion of the GUSB so that they can carry out their biological activity in the body. When there is an oversupply of free estrogen via estrogen signaling, this can lead to hyper-estrogenic pathologies such as endometriosis and cancer. On the other hand, reduced GUSB activity caused by dysbiosis can lead to hypo-estrogenic pathologies such as obesity, metabolic syndrome and cardiovascular and neurological diseases [23,198]. This association between the β-glucuronidases (GUSB) produced by the gut microbiome, as part of the estrobolome that reactivates estrogens, has been confirmed [199]. Unconjugated estrogens are transferred back into the bloodstream via the intestines by means of GUSB enzymes and can develop their hormonal effect elsewhere.

In addition to the estrogen-metabolizing gut microbiome, there is also a microbiome in the gut, as mentioned earlier, that metabolizes tryptophan instead of estrogens. This microbial community, which also includes *Klebsiella spp*., does not compete with the host cells of the intestine for tryptophan and therefore promotes the production of melatonin. The gastric and intestinal cells and the endogenous metabolism in the liver produce melatonin as a secondary metabolite via pre-beta-lipoproteins (VLDL) and LDL [200]. A well-known effect of melatonin is that it regulates the circadian rhythm. In addition, it shows oncostatic effects such as the inhibition of proliferation and stimulation of apoptosis, immunomodulation and anti-inflammatory effects; furthermore, it shows supportive effects for the anti-oxidation systems. In addition, it regulates blood vessel formation [201]. The background to this is that ER signaling is blocked by melatonin, which is analogous to the effects of a selective estrogen receptor modulator (SERM) [202,203]. However, melatonin also shows the properties of a selective estrogen enzyme modulator (SEEM) [204]. Thus, melatonin inhibits the enzymatic activity of estrone (E1) sulfatase, which converts estrone sulfate (E1S) into estrone (E1), but also the activity of 17β-hydroxysteroid dehydrogenase type 1 (17β-HSD1), which is responsible for the conversion of E1 to E2. This subsequently reduces the circulating estradiol levels in the plasma [205]. Melatonin also promotes the sulfation of estradiol to the inactive form of estrogen, estrogen sulfate (E1S), via the activity of estrone sulfotransferase (E1-ST), and thus shows an anti-estrogenic effect. The effects of estradiol (E2) are diverse; they promote, in addition to growth, e.g., the cervical epithelium, the immunosuppression in the microenvironment of the intraepithelial cervical lesions via their genomic effect on the estrogen receptor alpha (ERα). This also affects the cells responsible for immunosuppression such as the regulatory T cells (Tregs), the myeloid-derived suppressor cells (MDSCs) and cancer-associated fibroblasts (CAFs) [15] (Figure 3). These effects of E2 now bring us to the next subsection of our review, estrogen signaling.

### 3.2. Estrogen Signaling

Estradiol signals in a ligand-dependent and classical manner via the estrogen receptors α and β (ER α/β) and the G protein-coupled estrogen receptor 1 (GPER1), also known as G protein-coupled receptor 30 (GPR30). Independently, ligand signals in a non-classical way via other receptors such as the epidermal growth factor receptor (EGFR), the insulin growth factor receptor (IGFR) and the fibroblast growth factor receptor (FGFR) [206]. In addition, a genomic pathway is distinguished from a non-genomic pathway. In the genomic pathway, the hormone-receptor complexes are translocated to the nucleus, where the receptor dimers bind to estrogen-responsive elements (EREs) on the target gene promoter, triggering gene activation and epigenetic changes. The estrogen receptor serves as a transcription factor that regulates gene expression and thus cell proliferation and cell survival. Tissues that respond to fluctuating estrogen levels may experience organic changes [23]. As already mentioned in the last subchapter, one should not underestimate the indirect effects of estrogen signaling on the tumor microenvironment, such as the infiltrating immune cells, stroma cells and cancer-associated fibroblasts (CAFs) [206].

### 3.3. Influence of Estrogen Signaling on the Microenvironment of Cervical Intraepithelial Lesion, Cervical Pre-Cancer and in Tumor

Of all the work on cervical carcinogenesis, animal models have made their decisive contribution to elucidating the mechanisms of the course of infection with the high-risk HPV types and the resulting transformation of the cervical lesions. In a frequently cited work by Arbeit et al. from 1996 [207], the authors investigated the processes of carcinogenesis in a transgenic mouse model (K14-HPV16-E6/E7), which was permanently exposed to estradiol [207]. As already mentioned, chronic unresolved infection with one of the high-risk HPV strains is necessary for the start of the malignant transformation. However, further supporting co-factors, such as estradiol, are required for the full development of invasive carcinoma [208,209,210]. Sirtuin-1 (SIRT-1) cooperates with estrogen signaling in breast cancer tumorigenesis and progression [211]. SIRT-1 is also up-regulated in cervical carcinogenesis and leads via deacetylation to the destabilization of the tumor suppressor protein Werner syndrome protein (WRN). In HPV16 cervical cells, the destabilization of WRN resulted in increased basal cell proliferation, damage to DNA and epithelial thickening. This led to increased DNA replication via E1 and E2 and, as a result, to an increase in the proliferation of keratinocytes and the HPV life cycle in the lesions of the cervical neoplastic tissue [212]. It is also assumed that the likely use of birth control drugs and multiple maternities increases the risk of developing cervical squamous cell carcinoma (SCC) [213,214,215,216,217]. The association between viral infection and the effects of cofactors such as hormonal contraceptives in cervical carcinogenesis was examined by Marks MA et al. [218]. Analyzing cervical secretions from elderly women with HPV infection it was found that markers associated with anti-inflammation and allergies (IL-5, IL-9, IL-13, IL-17, EOTAXIN, GM-CSF and MIP-1α) were increased compared to HPV-negative women. In addition, T cell cytokines were shifted from interleukin 2 (IL-2) towards Eotaxin. This suggests immunosuppressive T cell responses via type 2 T helper cells (TH2 lymphocytes) [219]. Since estradiol, together with other factors, seems to suppress the immune response in cervical carcinogenesis, it is important to determine the estradiol concentrations in the blood plasma but also in the cervical secretions, namely the cervical mucus in postmenopausal HPV-infected women. During pregnancy, elevated levels of estradiol are found in blood plasma. A study from Denmark found a higher rate of mortality with cervical cancer during pregnancy or shortly after pregnancy [220].

#### 3.3.1. Estrogen Distribution in the Tumor and the Surrounding Microenvironment

Estrone (E1) and/or estradiol (E2) are delivered to the tumor and its microenvironment via the bloodstream or there is an increased local production and retention of the hormones within the tumors. The latter occurs via the increased activity of the cytochrome P450 enzyme, aromatase (CYP19A1), which converts androstenedione and testosterone into E2 [221]. Estradiol levels have been shown to increase in the tumor microenvironment (TME) of cervical carcinoma while present at regular levels in blood plasma. In the transformed cervical keratinocytes, estradiol was distributed within the cytoplasm and in the infiltrating immune cells, and in the stroma in both the cytoplasm and the nucleus [222,223]. Compared to estradiol levels, aromatase showed an analogous distribution, suggesting that the hormone is synthesized within the tumor microenvironment and tumor [222,224]. As an alternative to the synthesis pathways via aromatase, estradiol can also be synthesized in the tissue via other pathways. Estrone sulfate (E1S) is converted into estrone by means of estrone sulfatase (E1-STS) and then further into estradiol (E2) by means of the enzyme 17β-hydroxysteroid dehydrogenase type 1 (17β-HSD1). The two enzymes E1-STS and 17β-HSD were detected in vitro in the HPV-positive cervical carcinoma cell line Hela, but not in vivo or in xenografts from patients [194,225].

#### 3.3.2. Different Estrogen Signaling Pathways Causing Both, Pro- and Anti-Tumorigenic Effects in HPV-Positive Lesions of the Cervical Epithelium

Depending on the respective concentration of estrogens in the tissue of the cervix, there is a proliferation of the cancer cells and the suppression of their apoptosis induction at physiological hormone levels [226,227,228,229]. On the other hand, an opposite effect can also set in with a higher distribution of estradiol in the tissue, which disrupts protein translation on the ribosomes and leads to apoptotic cancer cell death [230,231]. The latter pathway, at high hormone concentrations, via estradiol-induced phosphodiesterase 3A (PDE3A) activation and transactivation of Schlafen, a family member 12 (SLFN12) protein that is predicted to act upstream or within the negative regulation of cell proliferation, leads to the inhibition of mitochondrial activity and the blockade of the apoptotic proteins Bcl2 and Mcl1 for cancer cell apoptosis [232].

In addition to the need for E2, the transgenic mouse models K14-HPV16/HPV18-E6/E7 were also dependent on the expression of the estrogen receptor α (ERα) [207,233]. It was not surprising that selective estrogen receptor modulators (SERMs) and selective estrogen receptor disruptors (SERDs) inhibited the growth of precancerous lesions and carcinomas in animal models [207,209,234,235,236,237]. It was surprising to see that in the transformed cervical keratinocytes from healthy tissue in the direction of malignant development, the expression of ERα was gradually lost, and in mature cancer, no ERα expression could be detected, while, in contrast, the expression of ERβ was preserved and both ERα and ERβ expression were determined in the surrounding stroma [238,239,240,241,242,243]. If tumor cells cannot use estradiol via the genomic pathway, the estradiol originating from the tumor microenvironment can also signal in the tumor cell via non-genomic pathways [206]. The pro-tumor and anti-tumor effects of estradiol on malignant lesions due to HPV infection are not contradictory [13]. Since carcinogenesis always depends on the interactions between the developing malignancy and its tumor microenvironment [27,121], we must focus our attention primarily on the bidirectional crosstalk between the tumor and its tumor microenvironment. These primarily include the HPV-infected keratinocytes of the cervical lesion, but also the immune cells infiltrating the tumor stroma, which play a key role in deciding the fate of the lesion towards malignancy [237,244,245]. We will now discuss the hormonal effects on the tumor stroma and its effects on the infiltration of immune-modulating cells of the immune response (Figure 3).

#### 3.3.3. The Importance of the ERα for the Tumor Stroma and the Tumor Microenvironment in Relation to the Development of Precancerous Lesions up to the Invasive Form of Cervical Carcinoma

Many benign cells of the cervical stroma express the estrogen receptor α. On the other hand, adequate estradiol levels are not produced in the blood plasma of women who are currently in their menstrual phase. This suggests that the hormone levels appropriate for ERα expression are produced by the hormone-sensitive tissues themselves [238,241,242]. The estrogen receptor α was expressed in 30% to more than 50% of the stromal cells surrounding the tumor from precancerous lesions and invasive cervical carcinoma, but not in the tumor itself. Immunohistochemistry showed inequality in the distribution of the receptor in the tumor stroma. It was present in all tumors, regardless of cancer grade, in the intracellular spaces between tumor cells in cancer-associated fibroblasts (CAFs), MDSCs and other subtypes of lymphocytes. The tumor cells themselves, on the other hand, did not show any ERα expression [222,223,242,246,247,248]. This relationship between the estrogen signaling of cervical squamous tumor cells and the surrounding stromal cells could only be explained by a paracrine delivery of estrogen signaling-derived signals from the stromal cells. In fact, a special procedure applied within the related transgenic mouse models helped to remove the ERα only from the stromal cells in order to demonstrate these paracrine mechanisms of estrogen signaling [239,249]. In addition to the results from the animal models, it was shown, in the human model of cervical carcinoma, that cancer-associated fibroblasts (CAFs), which were cultivated ex vivo, were estrogen receptor α positive. Thus, ERα genomic signaling in the CAFs induced the secretion of soluble molecules, which, in a paracrine manner, directly benefited malignant cell proliferation and migration, vascular angiogenesis and the epithelial–mesenchymal transition (EMT) of the metastatic tumor, and indirectly supported inflammatory processes within the cervical lesions that promoted cervical carcinogenesis [223]. Treating CAFs with a selective estrogen receptor modulator (SERM), in this case, methylpiperidinopyrazole (MPP), or a selective estrogen receptor disruptor (SERD), in this case, fulvestrant (Faslodex, ICI-182780), suppressed cell cycle- and metabolism-related genes in their expression. This attenuated tumorigenesis and angiogenesis as well as the functionality of estrogen signaling [223]. However, since the stromal genes, which are up-regulated via ERα signaling, are extremely important for cervical carcinogenesis [223,237,239,250], this implicates, conversely, that these genes should be therapeutically targeted [251].

#### 3.3.4. Estrogen Signaling in the Infiltrating Cells of the Immune Response in Cervical Carcinogenesis

Since the cervical epithelium slowly loses its ERα expression in the course of carcinogenesis via the various stages of cervical intraepithelial neoplasia (CIN1–3) to invasive CxCa, estradiol (E2) likely initially acts via the classical genomic pathway, with decreasing ERα-Expression levels within the transformed cells possibly via the non-classical, non-genomic pathways. Furthermore, E2 promotes an anti-inflammatory and regulatory immune microenvironment, which contributes decisively to the success of cervical carcinogenesis [218] (Figure 3).

Erythrocytes, granulocytes, monocytes and thrombocytes are formed during the myeloid hematopoiesis that takes place in the bone marrow. Estrogens induce this process and also enhance the mobility of MDSCs and their inherent immunosuppressive character [252]. In pregnant women, it has been shown that the increased estradiol levels in the blood plasma caused by pregnancy were sufficient to stimulate myeloid hematopoiesis and increase the mobility of MDSCs from the site of origin, the bone marrow, towards the spleen and local tumors. The ERα signaling of granulocytic myeloid suppressor cells (GrMDSCs) contributed to potentiating the level of immunosuppression within the tumor. This resulted in the progression of cervical carcinoma tumor cells that were ERα-negative on their own [246]. Ex vivo and in an orthotopic animal model of cervical carcinoma, the estrogen receptor antagonist fulvestrant was shown to be able to abrogate the immunosuppressive function of the tumor-infiltrating GrMDSCs [246].

In addition to the MDSCs, the regulatory T cells (Tregs or suppressor T cells) also suppress the immune response by infiltrating the tumor. Estradiol causes the expansion of the Tregs and induces the FOXP3 gene promoter, which is mainly responsible for the regulation of Tregs in mice [253,254]. However, it has been demonstrated that estradiol occurs in the tumor, its stroma and the infiltrating immune cells in human CxCa [222,224]. In comparison to other cells of the immune system, the intracellular estradiol levels were highest in the circulating, intratumoral Tregs and the Tregs of the draining lymph nodes [222]. In addition to the induction of the FOXP3 gene promoter, the associated FOXP3 expression and the maintenance of the control function of the Tregs, the immunosuppressive TGF-β and IL-10 cytokines were also released via the cell contact of the immunosuppressive Tregs. This was all controlled via ERα signaling [222]. It was not surprising that eight possible estrogen-responsive elements (EREs) were found in the FOXP3 locus of Foxp3+ regulatory T cells (FOXP3+ Tregs). The complex of the estrogen receptor α and estradiol was able to bind the translated FOXP3 protein product in the Tregs in humans [222]. Next to this classic genomic pathway of estrogen signaling, it could also be shown that Tregs could also signal via non-genomic (fast) signaling via the G protein-coupled estrogen receptor 1 (GPER1, GPR30) or the membrane-bound ERα. This led, via protein kinase B (Akt/PKB) phosphorylation, to the activation of programmed cell death protein 1 (PD1) signaling and/or increased expression of perforin. Perforin, a cytolytic protein, is found in the granules of cytotoxic T cells and NK cells, but also in Tregs. After degranulation, the cell membrane of the target cell is perforated. A pore is formed (hence the name perforin). Granzyme B enters the target cell and subsequently causes apoptotic cell death [255]. Thus, Tregs encountering cytotoxic T cells or NK cells in the tumor microenvironment could use the same mechanism to induce their apoptotic death by granzyme B and perforin, thus suppressing the immune response. Granzyme B and perforin are thus important for both the clearance of the malignant transformed tissue and its progression in vivo, and help to determine whether the pro- or anti-tumorigenic forces of the immune microenvironment fight for dominance within the cervical intraepithelial lesions and the tumor and its microenvironment prevail [255,256,257,258,259,260].

In contrast, selective estrogen receptor disruptors (SERDs) such as ICI 182,780 (fulvestrant, 7α,17β-[9-[(4,4,5,5,5-pentafluoropentyl)sulfinyl]nonyl]estra-1,3,5(10)-triene-3,17-diol) or RU 58,668 (11β,17β)-11-[4-[[5-[(4,4,5,5,5-pentafluoropentyl)sulfonyl]pentyl]-oxy]phenylestra-1,3,5,(10)-triene-3,17-diol) were used as antagonists of estrogen signaling. This resulted in the abrogation of the effects of estradiol on tumor-infiltrating Treg cells (CD4+CD25highCD127low) and the concomitant destruction of ERα and suppression of FOXP3 expression to terminate the suppressive effects of Tregs on both CD8+ and CD4+(CD4+CD25int) effector T cell subsets [222,261].

Another important type of immune cell is the M2 tumor-associated macrophages (M2-TAMs). These likewise immunosuppressive, tumor-promoting cells of the innate immune system are stimulated, at least in breast, ovarian and lung carcinomas, via the effect of estradiol to migrate into the tumors and secrete VEGF. The latter leads to positive feedback that prompts M2-TAMs to infiltrate the tumors in even larger numbers [206]. The proteinase inhibitor 9 (PI9) expression is increased via estrogen signaling in the immune cells, which suppresses the secretion of granzyme B (GrB) both endo- and exogenously [262,263]. The expression levels of the granzyme gene family are also down-regulated by the interaction of the HR HPV16 E7 oncoprotein with estrogen signaling [264]. Granzyme B is also secreted by keratinocytes. This suggests that estrogen signaling inhibits granzyme B expression via a similar effect in HPV-infected keratinocytes of the cervical epithelium in collaboration with HPV oncoproteins [265]. As we mentioned earlier, perforin and granzyme B promote apoptosis [255]. Granzyme B also promotes the degradation of collagen, an extremely important component of the extracellular matrix (ECM). This mechanism paves the way for the infiltration of the tumor microenvironment by cytotoxic T lymphocytes [266,267]. However, since granzyme B expression is now down-regulated via the collaboration of E2 with the HR HPV16 E7 oncoprotein, it saves the developing transformed keratinocyte from apoptotic cell death and protects it by preventing the invasion of effector T cells into the premalignant lesion prior to recognition and elimination by the immune system [263,268]. Chemokines such as CC chemokine ligand 2 (CCL2) and CCL5 promote tumor progression. Their expression is also induced by the action of estradiol, at least in breast cancer [269]. Both CD8+ cytotoxic effector T lymphocytes (CTLs), the NK cell mobilizing CD4+ T lymphocytes (T helper cells) and the regulatory T cells (Tregs) show estrogen receptor α (ERα) expression, but Tregs, compared to the other lymphocyte subgroups, show a higher ERα expression [222].

### 3.4. The Effect of ER Antagonists in Modulating the Immune Microenvironment of the Premalignant Cervical Lesion and CxCa

The use of selective estrogen receptor disruptors (SERDs, ER antagonists, ICIs) has shown to counteract the effects of estradiol on estrogen signaling and subsequently on the immune response cells infiltrating the tumor and its stroma, including cancer-associated fibroblasts (CAFs), MDSCs and regulatory (Th2) T lymphocytes (Tregs) in cervical carcinoma [209,222,223,246].

As already mentioned, estradiol (E2) acts both classically and ligand-dependently via the classical ERs and membrane-bound receptors such as GPER1 (GPR30), and non-classically and ligand-independently via other receptors such as EGFR, IGFR and FGFR. This is also dependent and independent of FOXP3 expression and dependent and independent of the programmed cell death 1 ligand 1 (PD-L1)/programmed cell death protein 1 (PD1) pathway [253,256,257,258,260]. When using ICIs such as fulvestrant, under the action of exogenously supplied E2, after some time there was a lifting of the suppression of ER signaling and a renewed secretion of cytokines, probably via signals transmitted via extranuclear activity pathways (RAS/MAPK and PI3K/AKT). This happened non-canonically, and further, e.g., via transcription factors such as the activating protein-1 (AP-1) and the specific protein-1 (Sp1) [183,222,270,271,272]. Given the variety of pathways in which effects can be exerted via estrogen signaling, it might make more sense to tackle the problem of estrogen signaling inhibition “at the root”, which is hormone synthesis. In addition to therapy with ER antagonists, the use of a group of anti-estrogens such as aromatase inhibitors (AIs) could offer a more effective approach in the therapy of CxCa. In a study on patients with breast cancer, it was found that continued therapy with aromatase inhibitors subsequently also reduced the incidence of malignant changes in the cervix. A 10-year incidence of high-grade cervical dysplasia (CIN2–3) was found in the group that has been screened regularly (HR = 0.49; 95% CI, 0.27 to 0.90; *p* = 0.0212), especially in women over 50 years of age (HR = 0.34; 95% CI, 0.14 to 0.80; *p* = 0.014). The protective effect of tamoxifen monotherapy against low-grade cervical dysplasia (CIN1) was found only in young women [273]. In another study, the main load of CxCa (43%) was found in postmenopausal women who also showed a high E2 concentration in the tumor [222]. This reinforces the importance of inhibition of local synthesis of E2 by AIs in the affected tissue [273]. Letrozole and anastrozole, two drugs from the group of AIs, led to a reduction in Th2 cytokines and an increase in Th1 cytokines as well as a simultaneous reduction in forkhead box protein P3 (FOXP3+) regulatory T cells (Tregs) in animal experiments as well as in humans [274,275]. Thus, the inhibition of E2 synthesis via the action of the AIs led to the reactivation of the immune function of (Th1) cytotoxic T lymphocytes (CTLs) [256,276] and the natural killer (NK) cells [268] within the tumor microenvironment. AIs abrogate the function of aromatase only between the conversion of androstenedione to estrone (E1) and the conversion of testosterone to estradiol (E2). It is important to remember that, especially in postmenopausal women, E2 is also synthesized via the conversion of the predominant form of the hormone in these women from estrone sulfate (E1S) to E1 by estrone sulphatase and further by type 1 17β-hydroxysteroid dehydrogenase (17β-HSD, HSD17B) [15,194,277,278] (Figure 4).

## 4. Conclusions and Prospects

Perhaps the most important difference in the carcinogenesis of cervical cancer, compared to other hormone-sensitive cancers such as colon, breast, endometrial, lung and prostate cancer, is the infection with human papillomavirus. In particular, the high-risk HPV types HPV16 (50% of cases) and HPV18 (20% of cases) are responsible [279,280]. HPV specializes in infecting mucosal membranes, such as the mucosal cervical epithelium. In the malignant progression of the cervical epithelium, metabolic and immune signaling are complemented by hormone signaling as the third level of complexity. They all affect the interactions between HPV and the infected keratinocytes, the invading regulatory immune cells, the tumor stroma, the cancer-associated fibroblasts (CAFs) and the endothelial cells of the blood and lymphatic vessels supplying the tumor. The crosstalk between the HPV-infected keratinocytes, estrogen signaling and immune cells, especially the innate immune system, not only determines the successful proliferation cycle of the virus, but also promotes the integration of the virus DNA into the host genome through endocrine, intracrine and paracrine signaling, eventually causing subsequent carcinogenesis. One has to make a distinction between transient and non-transient states in the possibly protracted fight of the immune system against HPV infection. The simultaneous occurrence of both pro- and anti-inflammatory cytokines at the beginning of the infection shows the importance of the mediators of the local immune system, which, via the effect of the HPV oncogenes, but also via hormone signaling, lead to general and/or specific immune suppression and thus result in immune evasion of the viral infection. The estrogen signaling, e.g., via GPER1 and ER, is of significant importance, since it not only inhibits apoptosis by increasing Bcl2, but also influences cell cycle progression via PI3K/Akt signaling and the expression of c-Myc and Cyclin D1 and also fuels the metabolism by amplifying the Warburg effect. HPV and the developing malignancy make use of both mechanisms, although “the days of the virus are numbered” the more the viral DNA integrates into the host DNA.

There is broader evidence for both a tumor-promoting and tumor-suppressing effect of estrogen signaling on carcinogenesis of cervical intraepithelial neoplasia. It has been shown that estrogen is even able to sensitize HeLa cervical carcinoma cells [230,231,232] and that ERα expression levels positively correlate with a patient’s overall survival (OS) [17,239,243,281,282,283,284,285,286,287,288,289,290]. Of course, the research results obtained in vitro cannot be transferred 1:1 to animal models or human studies. Not all variants of the estrogen receptors show the same effects, depending on the cell context. The variants of the ER, ERα and ERβ individually show similar effects on the innate immune system. However, they also act as antagonists [291]. The binding of estrogen to ERα-36, a truncated splice variant of ERα-66 on the ESR-1 gene, increases proliferation, migration and invasion of certain cell lines [292,293]. Many animal studies on the interaction between estrogen signaling and the consequences of HPV infection have been performed in the mouse transgenic model K14-E7 [294]. Although this estrogen-dependent model has provided important answers for deciphering the mechanisms of cervical carcinogenesis, the constitutive expressions of the E6 and E7 oncoproteins make it a non-ideal model, selecting for an immune microenvironment tolerogenic to E6 and E7. This does not correspond to the natural conditions in humans.

One must distinguish between the conditions in transient and chronic HPV infection, since transient infection lasts approximately 2.5 years before it is eliminated by the immune system, and inflammatory cytokines have already returned to their basal levels long before the infection is eliminated. This ensures limited tissue damage if the infection is inflammatory at all [19,20,295]. Levels of cytokines such as IL-10 are of limited value in determining the degree of immunosuppression within the cervicovaginal tissue, as this cytokine also serves to bind and deactivate IFN-γ to maintain regular homeostasis to restore the tissue. Next to the discrepancy between the cytokine levels in cervical mucus or in blood plasma and the conditions in situ, simultaneous infection with other pathogenic microorganisms such as *Chlamydia trachomatis* can also lead to increased expression of inflammatory cytokines and complicate the interpretation of whether the conditions indicate a more pro- or anti-inflammatory (immune-stimulating or immune-suppressing, respectively) microenvironment.

In the future, not only the interactions between the tumor and its tumor microenvironment (TME) must be examined in detail, but also between the individual members of the TME that infiltrate the tumor and thus shield it from immune recognition. In this context, it is also necessary to clarify how the expression of estrogen and its receptors is distributed in the individual components of the TME, since the expression levels of ERα within the tumor cells of the cervical carcinoma decrease sharply, and they are therefore dependent on the estrogen signaling of the TME. The fight against tumorigenesis must be fought on many levels. In addition to treating patients with selective estrogen receptor modulators (SERMs) and disruptors (SERDs) and aromatase inhibitors (AIs), immune checkpoint inhibitors (ICIs) can also be considered. The latter has shown good activity by inhibiting the binding between programmed cell death 1 ligand 1 (PD-L1) and its receptor, programmed cell death protein 1 (PD-1) (Nobel Prize in Medicine to James P. Allison and Tasuku Honjo in 2018), albeit with some serious side effects. However, these could be mitigated, for example, through the application of fecal microbiota transplantation (FMT) with bacteria such as *Akkermansia muciniphila*. Overall, it is not only for this reason that the healthy intestinal flora, which is able to maintain a woman’s vaginal health, is of particular importance [296,297,298,299]. Finally, we would like to point out the possibility of adoptive T-cell therapy (ACT), which has proven to be useful not only in the treatment of metastatic forms of cervical cancer [300,301,302,303,304]. In addition to surgical resection of the malignant tissue of the cervix and chemotherapy and radiation therapy, there are other options for treating intraepithelial lesions and more or less invasive types of cervical carcinoma with molecular means. In this review article, we have discussed the associated molecular principles and outlined perspectives. With all the backgrounds of the complex processes within the malignant process presented here, one should not only consider the individual components of the tumor microenvironment on their own. Rather, the therapy of cervical cancer requires a holistic concept that takes into account all processes in the HPV replication cycle, the different influences of metabolism and estrogen signaling on the development of cancer, the immune competence and the hormone or hormone receptor status of the woman and finally her microbial health status. Since we still have to consider the already complex situation in general carcinogenesis in the case of cervical carcinoma at the level of initiation by HPV infection, enough questions remain unanswered that still need to be clarified. Since not all women will be vaccinated against the most common HPV types in the foreseeable future and will also not be able to take part in appropriate screening programs, the topic of therapy for cervical carcinoma will remain very topical for most women.
